# Technologies for Supporting Individuals and Caregivers Living With Fetal Alcohol Spectrum Disorder: Scoping Review

**DOI:** 10.2196/51074

**Published:** 2024-07-11

**Authors:** Joanna Ting Wai Chu, Holly Wilson, Cynthia Zhiyin Cai, Jessica C McCormack, David Newcombe, Chris Bullen

**Affiliations:** 1 National Institute for Health Innovation, School of Population Health, The University of Auckland, Auckland, New Zealand; 2 Centre for Arts and Social Transformation, Faculty of Education and Social Work, The University of Auckland, Auckland, New Zealand; 3 Centres for Addiction Research, Medical and Health Sciences, The University of Auckland, Auckland, New Zealand; 4 Social and Community Health, School of Population Health, The University of Auckland, Auckland, New Zealand; 5 Sensory Neuroscience Lab, Food Science, University of Otago, Dunedin, New Zealand

**Keywords:** fetal alcohol, scoping review, technology, caregivers, diagnosis, support, intervention, fetal alcohol spectrum disorder, FASD, developmental disability, lifelong support, caregiver, accessibility, alcohol, alcohol intake, pregnant substance, pregnant, fetal, PRISMA, Preferred Reporting Items for Systematic Reviews and Meta-Analyses, mobile phone

## Abstract

**Background:**

Fetal alcohol spectrum disorder (FASD) is a common developmental disability that requires lifelong and ongoing support but is often difficult to find due to the lack of trained professionals, funding, and support available. Technology could provide cost-effective, accessible, and effective support to those living with FASD and their caregivers.

**Objective:**

In this review, we aimed to explore the use of technology available for supporting people living with FASD and their caregivers.

**Methods:**

We conducted a scoping review to identify studies that included technology for people with FASD or their caregivers; focused on FASD; used an empirical study design; were published since 2005; and used technology for assessment, diagnosis, monitoring, or support for people with FASD. We searched MEDLINE, Web of Science, Scopus, Embase, APA PsycINFO, ACM Digital Library, JMIR Publications journals, the Cochrane Library, EBSCOhost, IEEE, study references, and gray literature to find studies. Searches were conducted in November 2022 and updated in January 2024. Two reviewers (CZC and HW) independently completed study selection and data extraction.

**Results:**

In total, 17 studies exploring technology available for people with FASD showed that technology could be effective at teaching skills, supporting caregivers, and helping people with FASD develop skills.

**Conclusions:**

Technology could provide support for people affected by FASD; however, currently there is limited technology available, and the potential benefits are largely unexplored.

## Introduction

### Background

*Fetal alcohol spectrum disorder* (FASD) is a diagnostic term that describes the effects of prenatal alcohol exposure (PAE) on an individual [1]. Globally, it is estimated that approximately 7.7 of every 1000 births are affected by FASD [2]. People with FASD may experience restricted growth, diminished neurological and cognitive functioning, and behavioral problems [1]. These symptoms result in common disabilities, such as difficulties with memory, language, cognition, executive function, social skills, and attention [1]. People living with FASD face challenges with peer relationships, education, employment, independence [3,4], and mental health, including higher rates of suicide [5] and lower life expectancy [6].

Early interventions [3], including assessment, diagnosis, and effective support from a young age, are key to favorable outcomes [7]. Without such interventions, people with FASD are more likely to experience difficulties throughout their lifetime, such as school failure, poor mental health, and substance dependency [5,8]. However, accessing early interventions is difficult [9], and people can experience lengthy delays waiting for a diagnosis and support. Several factors contribute to these difficulties including a lack of FASD-informed and trained health professionals [10], lack of FASD-informed support, and lack of effective interventions available for those with FASD or their caregivers [11]. Technology could offer a low-cost and effective support to people with FASD.

The term *technology* encompasses a range of technologies; however, in this review, we limited technology to the delivery of support through virtual reality (VR), computer-based interventions, smartphone apps, artificial intelligence (AI), telehealth, computer games, wearable technology, or technologies that facilitate therapy or support. We use the National Institute for Health and Care Research (NIHR) [12] definition of technologies that include VR assessment or therapy, digital technologies, telehealth, computer-based assessment or therapies, real-time monitoring and wearable devices, smartphone apps, and sensors that can improve patient outcomes and health service efficiencies.

Innovations in technology hold promise to drive efficiencies, improve outcomes, and widen access to health care delivery [13]. Technology can support people’s health including aiding diagnosis and delivering effective support. Technology has improved diagnosis by facilitating the detection of neurocognitive impairments experienced by those with neurodevelopmental disorders. Technology has aided the screening of brain anatomy and activity and the screening of social and motor skill deficits [14,15]. Likewise, telehealth can provide specialist expertise to individuals living in remote communities [15,16], such as delivering FASD assessments to people living in remote Canada [17]. Digital interventions can support peoples’ mental health [18,19], while for those with intellectual disabilities, digital interventions can support well-being [20] and the development of key skills [21]. VR-delivered interventions can provide favorable outcomes for mental illness [18,19,22,23] and pain management [24] and improve emotional recognition for people with autism spectrum disorder (ASD) [25] and cognitive skills for those with attention-deficit/hyperactivity disorder (ADHD) [26]. These technologies have been found to be capable of delivering automated and self-directed support, at low cost without burdening health professionals, to patients and families with a range of conditions and disabilities [14,19]. Technology has been well received by people with intellectual disabilities [16,20,21,27], but the content needs to be adapted to the skills and needs of the intended population [27].

Despite the increasing adoption of technological advances in health care delivery, research on the application of such technologies used to support or assist people with FASD is sparse. A systematic review conducted on the implementation of technologies to assess, monitor, and treat neurodevelopmental disorders did not report on any FASD-related studies [28]. To date, no study has been conducted to review available technologies to support people living with FASD. Much of this research has focused on digital interventions that can reduce PAE rather than support for FASD [29]. A recent systematic review of screening tools for FASD identified several screening tools to support clinicians to diagnose FASD or fetal alcohol syndrome (FAS) [17], and telehealth has been explored for people with FASD [30]. However, the focus of the review was not on technology-based tools alone [17].

### Objectives

We conducted a scoping review to explore what technologies have been applied or implemented to support caregivers and people with FASD with assessment, monitoring, and support and to identify current gaps and limitations within the existing evidence base. Scoping reviews are particularly relevant to disciplines with emerging evidence where the existence of few randomized controlled trials makes it difficult for researchers to conduct systematic reviews. Another strength is that scoping reviews can include a range of study designs, in both published and gray literature, addressing questions beyond those related to intervention effectiveness [31].

## Methods

### Protocol and Registration

We conducted a scoping review following the PRISMA-ScR (Preferred Reporting Items for Systematic Reviews and Meta-Analyses Extension for Scoping Reviews) [32]. The review protocol was registered on PROSPERO (CRD42022364885).

### Eligibility Criteria

Studies were eligible if they met the following criteria: technology (modified and adapted from NIHR [28]) was intended for people living with FASD or their caregivers; focused on FASD; used an empirical study design (qualitative or quantitative); were published after 2005 (the year when the first FASD diagnostic criteria were made available); included digital technology designed to aid the assessment, diagnosis, monitoring, or support for people with FASD and involved direct interaction from the person with FASD or their caregiver; and were published in the English language. We excluded technology that did not require direct interaction by the person with FASD or their caregiver, such as tools designed to assist professionals; that were noninteractive and provided only written material, such as noninteractive websites; and if the focus was not FASD.

### Information Sources and Search Strategy

Between November 14 and November 30, 2022, we searched the literature to identify studies that examined technology to support people with FASD. We searched the following databases: MEDLINE, Web of Science, Scopus, Embase, APA PsycINFO, ACM Digital Library, JMIR Publications journals, the Cochrane Library, EBSCOhost, and IEEE. To find gray literature and publicly available technology, we searched the ProQuest Dissertations and Theses database, PROSPERO, ClinicalTrials.gov, World Health Organization, International Clinical Trials Registry Platform, Google Scholar (first 100 results), Apple App Store, and Google Play Store. Searches were rerun in April 2024. Of the studies that were included in the final analysis, we searched the reference list and identified no new references. The search terms combined FASD and technology-related terms (see Multimedia Appendix 1 for an example of a search strategy). For the gray literature, the search terms were modified when it was not possible to combine terms.

### Study Selection

Search results were extracted, and then duplicates were removed. Then, the title and abstract were independently screened based on the inclusion criteria by 2 reviewers (CZC and HW). Following this, we obtained the full text of all possible papers, and they were independently screened by 2 reviewers (CZC and HW).

### Data Charting

Data were extracted to a bespoke Microsoft Excel (Microsoft Corp) spreadsheet; one author (CZC) extracted data for all studies, and a random sample of 10% of papers was reviewed by another author (HW) to check reliability. The data extracted included author, date, full citation, country, number and characteristics of participants, description of technology, research approach, methods, data analysis technique, and main findings. Any disagreement between reviewers was resolved by consensus.

### Critical Appraisal of Individual Sources of Evidence

The methodological quality of each study was accessed using the Mixed Methods Appraisal Tool (MMAT) [33,34]. We chose to determine the quality of each study to explore the quality of literature, and all studies were included in the final analysis regardless of their quality. We used the MMAT, as the quality of different study designs can be assessed with the MMAT. The MMAT determines the quality of each study with two steps. First, each paper is screened to ensure the MMAT can be applied. Then, based on the study type, several questions determine the quality of the studies’ research approach, methodology, and results based on each criterion for different study types. Each study has 5 quality questions applied to determine the quality of each study, and no overall score is given, but rather each response is considered. Two authors (CZC and HW) independently applied the MMAT to each study, and any disagreements were resolved through discussion with all authors.

### Synthesis of Results

To synthesize technology available to support those with FASD, a narrative synthesis was conducted [35]. The main findings from each study were exported into NVivo (QSR International), where they were grouped by common technology for assessment, diagnosis, and support for FASD. A narrative synthesis was conducted, as it enables the inclusion of qualitative and quantitative findings and provides an overview of the existing technology available to support people with FASD [35].

## Results

### Selection of Sources of Evidence

**Figure 1.**
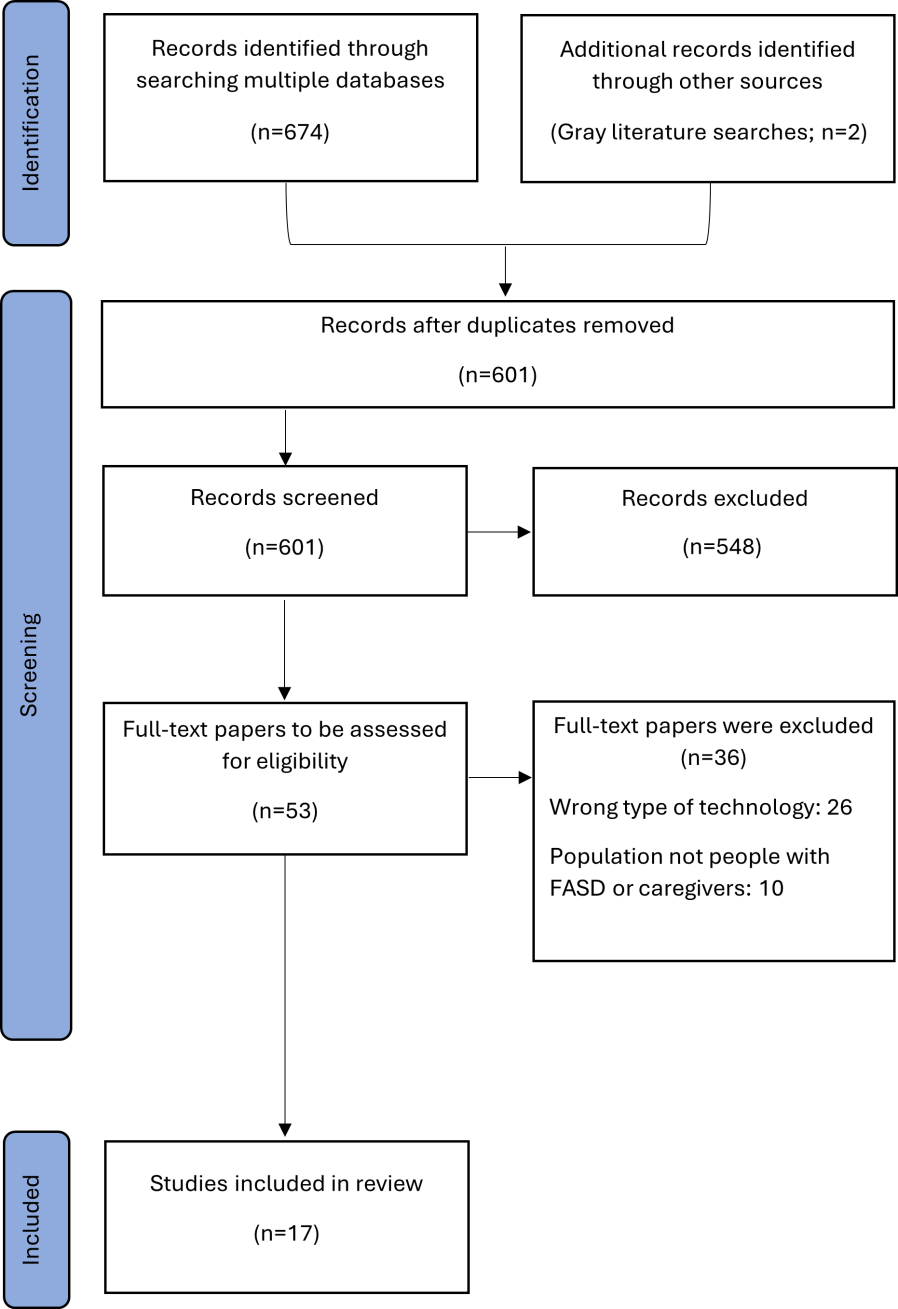
PRISMA (Preferred Reporting Items for Systematic Reviews and Meta-Analyses) flowchart of included studies. FASD: fetal alcohol spectrum disorder.

Our search produced 676 results. After duplicates were removed (n=75) and criteria were applied, 17 studies met the inclusion criteria and were included in this review (Figure 1).

### Characteristics of Sources of Evidence

In total, 17 studies [36-52] were included in this review. All studies explored the experiences of stakeholders with technology, but no studies included publicly available apps or technology. Of the 17 included studies, 15 studies [36-41,43-47,49-52] included participants; the remainder were study protocols. In total, 231 caregivers of people with FASD and 168 children participated. Of the 168 children, 157 (94%) had FASD, and 11 (6%) were typically developing. All children who participated were 18 years of age or younger, and of these, 97 (58%) were younger than 10 years of age. Most studies were conducted in the United States (n=10, 59%) [36,37,40,41,43-47,52], Canada (n=3, 18%) [38,39,48], and Australia (n=2, 12%) [49,50], with 1 conducted in South Africa (n=1, 6%) [42] and the United Kingdom (n=1, 6%) [51]. The included studies can be classified as those that evaluated the efficacy of a technology on an outcome (n=8, 47%) [36,37,40,41,43-45,52], focused on the design or implementation (n=7, 41%) [38,39,46,47,49-51], were study protocols (n=2, 12%) [42,48], and had no published follow-up studies. Of these studies that evaluated the efficacy (n=8, 47%), the study designs used were randomized controlled trials (n=5, 63%) [37,40,41,43,52], case studies (n=1, 12%) [45], and pre-post study designs (n=2, 25%) [36,44]. The type of technologies in these studies included web-based interactive platforms (n=3, 18%), VR-based technology (n=4, 23%), computer games (n=4, 23%), apps (n=2, 12%), and interventions delivered via technology (n=4, 23%).

### Critical Appraisal Within Sources of Evidence

The MMAT was applied to 15 of the 17 studies. The tool could not be applied to 2 because they were study protocols [42,48]. The results of the quality assessment are shown in Multimedia Appendix 2 [36-52]. Most (n=10, 71%) [36,38-41,44-47,49] of the studies meet all criteria. While 3 (18%) [37,43,52] studies only meet 2 or 3 of the criteria as the methods lacked sufficient information to determine the quality of the studies.

### Results of Individual Sources of Evidence

In total, 17 studies were included in this review, and 2 of these studies were study protocols (see Multimedia Appendix 3 [36-52] for characteristics of individual studies).

### Synthesis of Results

#### Overview

Our narrative analysis identified 2 main themes (assessment and diagnosis of FASD and support) and 2 subthemes within support (caregiver support and skill development). The majority of technology was designed to support people with FASD or their caregivers. Most studies focused on support, with only 1 study exploring both assessment and support.

#### Assessment and Diagnosis of FASD

Only 1 study explored the experiences of caregivers with technology to support the assessment and diagnosis of FASD. This study explored the perceptions of families of the use of telehealth-assisted assessment and diagnosis of FASD in remote communities in Manitoba, Canada [38]. The birth and adoptive caregivers who participated in the interviews were satisfied with the use of telehealth to aid the assessment processes and reduced barriers to access.

#### Support

##### Overview

All other studies focused on supporting people with FASD or their caregiver’s following diagnosis of FASD. Two main subthemes were identified: (1) caregiver support, where technology was designed to support the caregivers of people with FASD, and (2) skill development, where technology aimed to develop skills for people living with FASD.

##### Caregiver Support

Technology designed to support caregivers of people with FASD was used in 8 studies [39,40,46-51]. These studies explored 3 distinct technologies. First, Families Moving Forward Connect [46,47], an app designed to educate and support caregivers, provided resources and opportunities to connect with others with experience with FASD. These studies explored the design and feasibility of the app and found the app was received positively by caregivers, including the content of the app and the connection to others, but some participants reported difficulty with using the app.

Second, Strongest Families FASD [39,48], a web-based interactive program, was designed to promote parenting and reduce distress in caregivers through video or audio clips and interactive activities. One study explored the usability of the website through the experiences of biological and adoptive caregivers, who found the program to be easy to use and the content relevant [39]. The second study was a protocol study with no currently available outcomes [48]. Finally, a study explored the efficacy of web-based, in-person, and information-only formats in supporting the knowledge and behavioral regulation in caregivers of people diagnosed with FAS and partial FAS [40]. All 3 formats (online, in-person, and information) showed improvements in knowledge of behavioral regulation, but the online format showed no improvements in actual behaviors when the in-person and information formats did.

Finally, educational training programs delivered online were designed to support caregivers. The Salford Parents and Carers Education Course for Improvements in FASD Outcomes in Children (SPECIFiC) [51] is a 7-session educational program that was delivered online through videoconferencing. SPECIFiC was designed to be delivered in person; however, due to the COVID-19 pandemic, the program was delivered remotely. Getting on With It (GOWI) is a 6-session program delivered online via Zoom (Zoom Video Communications) that aimed to support caregivers of those with FASD [50]. GOWI was adapted from an in-person program [53] and delivered via Zoom. The original program consists of 1 session delivered over several weeks, each with a different topic. Some of the topics include an introduction to FASD and dealing with systems and getting on with professionals [53]. These programs then developed into Families Linking with Families, which is a 7-session program delivered online via Zoom [49]. Caregivers who completed GOWI and Families Moving Forward Connect were satisfied with the program and reported improvements in their knowledge of FASD [49,50].

### Skill Development

In total, 7 studies explored the use of technology to promote skill development for people with FASD. Within this subtheme, technology focused on the development of motor skills, life skills, disruptive behavior, and executive functioning.

#### Motor Skills

In total, 3 studies [43,44,52] explored the efficacy of Sensorimotor Training to Affect Balance, Engagement and Learning (STABEL) technology, a VR program that required children and adolescents with FASD to complete virtual tasks while the surface they were standing on changed. STABEL was designed to improve balance and motor skills for those living with FASD. STABEL was found to be engaging and showed improvement in motor skills for those with FASD who were assigned to the STABEL group relative to a control group.

#### Life Skills

In total, 2 studies [36,45] explored technology that focused on developing life skills, specifically fire and road safety, for children younger than 10 years of age with FASD. Both technologies required children to complete tasks in either a VR or computer-based game. The tasks focused on developing the relevant fire and road safety steps that would be translated into real-world settings. Across both studies, people with FASD effectively learned road and fire safety skills. However, when the road and fire skills were tested in real-world settings 1 week after receiving the technology, participants did not maintain the skills they had developed.

#### Disruptive Behavior

In total, 2 studies [37,41] explored the GoFAR program, a 3-component program aimed to reduce disruptive behaviors. In this program, children younger than 10 years of age with FASD use a computer game, where they navigate characters through a range of challenges designed to improve self-regulation. Therapy sessions are offered for the primary parents or caregivers of the children to educate them to promote self-regulation. Then, behavior analogy therapy is offered for the child and caregiver to support the implementation of behavior in real-life settings. The program was well received by caregivers and people with FASD. For individuals with FASD, the program showed improvements in behavioral skills and a reduction in disruptive behavior.

#### Executive Functioning

One protocol planned to test the efficacy of a game developed by the Foundation of Alcohol Related Research (FARR) known as the FARR game [42], a computerized cognitive training game in which people with FASD must navigate through more demanding cognitive tasks, designed to improve attention, memory, inhibition, and working memory. At the time of this review, no publication with outcomes for this protocol was available.

### Stakeholder Engagement

Of the studies that were included in the review, none involved caregivers or people living with FASD in the design or development process. Two studies sought caregiver input into the design and usability of an app and web-based program during the development of the technology [39,47]. However, the content and design of the app had been largely established prior to stakeholder engagement.

## Discussion

### Principal Results and Summary of Evidence

Although research is limited, there is growing evidence to show that technology can support skill development for people with FASD and support the well-being of caregivers of those with FASD. Of the few studies, most focused on developing skills for those with FASD and showed improvements in motor skills, life skills, disruptive behavior, and executive functioning. Technology has only been investigated in few of the neurocognitive domains PAE can affect, such as affect regulation and behavioral regulation [1]. A small number of studies have explored the use of technology to support caregivers. These studies showed that the technology (such as app, telehealth, and web-based programs) is well received and useful to caregivers. This review suggests that technology could be a viable option to support people with FASD and their caregivers, but currently technology is underused and remains largely unexplored.

### Assessment and Diagnosis of FASD

Evidence suggests that professionals feel unprepared to support and diagnose people with FASD [10,54] and can hold stigmatizing beliefs [55]. This stigma and lack of knowledge of health professionals can result in hesitancy to diagnose people with FASD and long wait times for a diagnosis. Significant delay between seeking help and a confirmed diagnoses of FASD can lead to poor outcomes for both the families and individuals. Technology potentially offers a way of improving the diagnostic pathway for FASD. For example, there is growing evidence supporting the potential for using telehealth and computer-based assessment methods to improve access to assessment and diagnosis of ASD [15]. Yet, the use of telehealth for those with FASD has only been explored in 3 studies [38,56,57], of which only 1 looked at the experiences of caregivers [38], which showed that telehealth can bring FASD diagnosis and support to those living in remote locations. Similarly, technology has been used to aid health professionals in diagnosing people with FASD [17]. Future research could explore the use of technology to facilitate self-screening of suspected FASD, such as detecting common difficulties experienced by those with FASD. If technology could screen for FASD, this could encourage the seeking of formal diagnosis, therefore facilitating a formal diagnosis.

There is potential for emerging technologies, like AI and machine learning, to be of use in assessment and diagnosis, although we did not find any examples in our scoping review. For example, combining facial recognition software and AI could be used to detect facial dysmorphia in FAS. Additionally, recent studies have made use of machine learning models to analyze brain imaging data to improve diagnosis and treatment outcomes for ADHD [58]. Telehealth solutions incorporating virtual assessments and consultations could provide FASD assessment, diagnosis, and support to those living in remote locations or underserved areas.

### Support and Interventions

People with FASD and their caregivers often experience difficulties accessing support due to a lack of support available, trained professionals [8,37], and funding to access services. Despite a rapid growth in the use of telehealth, computer-based therapies, and digital technologies to support physical and mental health [18,19], relatively few technologies have been designed specifically for FASD. As technology has showed promise in supporting those with intellectual disability [20,21,27], ASD [25], and neurodevelopmental disabilities [14], technology could provide effective support to those with FASD. For instance, telehealth has been used extensively in delivering health care remotely to those with intellectual disabilities [16]. Future research could explore the use of telehealth services to provide support for FASD to families living in remote areas or to enable the delivery of group-based parent training.

International reviews indicate that VR experiences can support people with anxiety [22] and depression [59] and support mental health for those with intellectual disabilities [20]. Specifically, VR programs have been used to improve attention and memory for people with ADHD [26] and emotional recognition for those with ASD [25]. Our review shows that VR can improve motor skills [43,44] and safety skills [45] for people with FASD, demonstrating the potential for VR technology to target specific skill development for those with FASD.

In addition, VR enables people to be immersed in a virtual world and situations, to experience situations to develop skills, and to experience the viewpoint of other people. VR can promote perspective taking and empathy and allow people to experience a condition, which in turn has the potential to reduce stigma toward those with mental illness [40], depression [41], and schizophrenia [13]. People with FASD can experience poorer mental health [5,38], in part driven by stigma [39], social isolation, and everyday difficulties people with FASD experience [7]. Professionals believe that FASD can be a highly stigmatized disability [54,55]. Therefore, VR experiences for professionals might promote understanding and reduce FASD stigma.

There is currently no published research on the use of real-time monitoring and wearable devices to support people with FASD. Wearable devices, such as those that monitor real-time body signals of heartbeat and respirations for stress level, have been used to capture stress response in daily life in children with ASD [60]. The findings can aid the development of real-time interventions and tailored treatment. A systematic review on wearables and mobile technologies interventions in ASD alone found 83 publications [61]. Compared with other neurodevelopmental disorders, we found the use of technological advances in FASD to be limited. Similar technology, wearable technology, VR experiences, and telehealth could support those living with FASD and their caregivers. Therefore, future research could adapt and co-design technology for those with FASD. The potential of technology for supporting individuals and caregivers living with FASD is largely unexplored. However, there may be challenges to adopting these technologies, such as cost of equipment, training requirements, and issues with data quality [62,63].

### Support Across the Life Span

The challenges people with FASD experience persist throughout their lifetime [9]. Those who are not provided with adequate and early support are likely to experience challenges with employment, independence, living situations, and forensic engagement [3,8]. In this review, no technology or studies explored the use of technology to support people older than 18 years of age. FASD research rarely focuses on those older than 18 years of age living with FASD [11]. This gap is problematic, given that people with FASD require lifelong support, and with very little support and trained professionals available, technology could provide a cost-effective and potentially effective avenue to support people with FASD.

Given the potential of technology to support those with FASD and their caregivers, there is a need to advocate for policy changes to investigate, develop, and sustain technology for those with FASD. Digital platforms could be used to facilitate collaboration between policy makers, researchers, health care providers, and community stakeholders to develop evidence-based policies and allocate resources. By focusing on policies and solutions, researchers, practitioners, and policy makers can harness the potential of digital solutions to improve the prevention, diagnosis, intervention, and support for individuals affected by FASD, ultimately enhancing their quality of life and well-being.

### End-User Involvement

Technology designed for caregivers and people with FASD could be best created through consultation and feedback from those whom the technology is intended for; however, only 2 studies in our review sought feedback from end users in the development and only after the material had largely been finalized. Evidence consistently suggests the importance of stakeholder engagement during the development process [64,65]. People with FASD can face unique challenges with executive functioning, memory, and attention [1], which could affect their ability to engage with and gain benefits through technology. The diversity of individuals with FASD must also be considered when developing technological tools. If people with FASD and their caregivers are not considered in the development of technologies, it could drive health inequities, as the support and interventions are less likely to be accessible to people with FASD. Therefore, it is important to involve and respond to stakeholder voices at all stages of developments to increase the uptake and usability of technology. Future research needs to explore the accessibility of technology, feasibility of types of technologies, and the type of technology solutions that would support those with FASD. Identifying the views of those with FASD on technology can provide insight into the main ethical issues of concern and could lead to effective solutions and supports that could be scaled up.

### Limitations

Our review may be limited through the databases and search terms that were used. We used a broad definition of technology to capture the wide-ranging and rapidly advancing field. However, our definition of technology may have limited our searches to identify different types of technology. A scoping review enables us to include unpublished literature and publicly available technology such as through the app store. As with any scoping review, we are unable to examine the effectiveness of technology for those with FASD but rather just explore the scope of the existing technology.

It should also be noted that technology might be used in clinical practices, but in this review, we have found limited published evidence about efficacy, costs, or user impact. Technology is an area of rapid development, and there are likely barriers between the use of technology in research and its use clinically. Future research, such as pragmatic trials and service evaluation to evaluate the real-world impact of technology in clinical practices, is needed.

### Conclusions

The potential for technology to provide vital support to people living with FASD and their caregivers is largely unexplored. Present examples are limited to specific skills and caregiver support. Learnings can be drawn from the ASD field where transformative technological advances have been examined and applied to those with ASD in clinical and real-world practices. There are areas for development of tools and technology to promote skill development, connection to others affected by FASD, and well-being and provide cost-effective diagnosis, assessment, and support for the those affected by FASD. The value of technologies is yet to be applied in any area of FASD, despite a growing demand for more effective and efficient services. There is an urgent need to explore the opportunities offered by technology to support those living with FASD.

## Supplementary material

10.2196/51074 Multimedia Appendix 1 Cochrane Library search strategy.

10.2196/51074 Multimedia Appendix 2 Quality assessment of each study.

10.2196/51074 Multimedia Appendix 3 Characteristics of included studies.

10.2196/51074 Checklist 1 Preferred Reporting Items for Systematic Reviews and Meta-Analyses Extension for Scoping Reviews (PRISMA-ScR) checklist.

## Abbreviations

ADHD: attention-deficit/hyperactivity disorder
AI: artificial intelligence
ASD: autism spectrum disorder
FARR: Foundation of Alcohol Related Research
FAS: fetal alcohol syndrome
FASD: fetal alcohol spectrum disorder
GOWI: Getting on With It
MMAT: Mixed Methods Appraisal Tool
NIHR: National Institute for Health and Care Research
PAE: prenatal alcohol exposure
PRISMA-ScR: Preferred Reporting Items for Systematic Reviews and Meta-Analyses Extension for Scoping Reviews
SPECIFiC: Salford Parents and Carers Education Course for Improvements in FASD Outcomes in Children
STABEL: Sensorimotor Training to Affect Balance, Engagement and Learning
VR: virtual reality

## References

[1] Cook JL, Green CR, Lilley CM (2016). Fetal alcohol spectrum disorder: a guideline for diagnosis across the lifespan. CMAJ.

[2] Lange S, Probst C, Gmel G, Rehm J, Burd L, Popova S (2017). Global prevalence of fetal alcohol spectrum disorder among children and youth: a systematic review and meta-analysis. JAMA Pediatr.

[3] Streissguth AP, Bookstein FL, Barr HM, Sampson PD, O’Malley K, Young JK (2004). Risk factors for adverse life outcomes in fetal alcohol syndrome and fetal alcohol effects. J Dev Behav Pediatr.

[4] Rangmar J, Hjern A, Vinnerljung B, Strömland K, Aronson M, Fahlke C (2015). Psychosocial outcomes of fetal alcohol syndrome in adulthood. Pediatrics.

[5] Flannigan K, McMorris C, Ewasiuk A (2022). Suicidality and associated factors among individuals assessed for fetal alcohol spectrum disorder across the lifespan in Canada. Can J Psychiatry.

[6] Thanh NX, Jonsson E (2016). Life expectancy of people with fetal alcohol syndrome. J Popul Ther Clin Pharmacol.

[7] Popova S, Lange S, Burd L, Chudley AE, Clarren SK, Rehm J (2013). Cost of fetal alcohol spectrum disorder diagnosis in Canada. PLoS One.

[8] McLachlan K, Flannigan K, Temple V, Unsworth K, Cook JL (2020). Difficulties in daily living experienced by adolescents, transition-aged youth, and adults with fetal alcohol spectrum disorder. Alcohol Clin Exp Res.

[9] Domeij H, Fahlström G, Bertilsson G (2018). Experiences of living with fetal alcohol spectrum disorders: a systematic review and synthesis of qualitative data. Dev Med Child Neurol.

[10] McCormack JC, Chu JTW, Marsh S, Bullen C (2022). Knowledge, attitudes, and practices of fetal alcohol spectrum disorder in health, justice, and education professionals: a systematic review. Res Dev Disabil.

[11] Reid N, Dawe S, Shelton D (2015). Systematic review of fetal alcohol spectrum disorder interventions across the life span. Alcohol Clin Exp Res.

[12] (2024). National Institute for Health and Care Research.

[13] Abernethy A, Adams L, Barrett M (2022). The promise of digital health: then, now, and the future. NAM Perspect.

[14] Ribas MO, Micai M, Caruso A, Fulceri F, Fazio M, Scattoni ML (2023). Technologies to support the diagnosis and/or treatment of neurodevelopmental disorders: a systematic review. Neurosci Biobehav Rev.

[15] Alfuraydan M, Croxall J, Hurt L, Kerr M, Brophy S (2020). Use of telehealth for facilitating the diagnostic assessment of autism spectrum disorder (ASD): a scoping review. PLoS One.

[16] Selick A, Bobbette N, Lunsky Y, Hamdani Y, Rayner J, Durbin J (2021). Virtual health care for adult patients with intellectual and developmental disabilities: a scoping review. Disabil Health J.

[17] Lim YH, Watkins RE, Jones H, Kippin NR, Finlay-Jones A (2022). Fetal alcohol spectrum disorders screening tools: a systematic review. Res Dev Disabil.

[18] Garrido S, Millington C, Cheers D (2019). What works and what doesn't work? A systematic review of digital mental health interventions for depression and anxiety in young people. Front Psychiatry.

[19] De Witte NAJ, Joris S, Van Assche E, Van Daele T (2021). Technological and digital interventions for mental health and wellbeing: an overview of systematic reviews. Front Digit Health.

[20] Oudshoorn CEM, Frielink N, Nijs SLP, Embregts P (2021). Psychological eHealth interventions for people with intellectual disabilities: a scoping review. J Appl Res Intellect Disabil.

[21] Oudshoorn CEM, Frielink N, Nijs SLP, Embregts P (2020). eHealth in the support of people with mild intellectual disability in daily life: a systematic review. J Appl Res Intellect Disabil.

[22] van Loenen I, Scholten W, Muntingh A, Smit J, Batelaan N (2022). The effectiveness of virtual reality exposure-based cognitive behavioral therapy for severe anxiety disorders, obsessive-compulsive disorder, and posttraumatic stress disorder: meta-analysis. J Med Internet Res.

[23] Kalyanaraman SS, Penn DL, Ivory JD, Judge A (2010). The virtual doppelganger: effects of a virtual reality simulator on perceptions of schizophrenia. J Nerv Ment Dis.

[24] Dreesmann NJ, Su H, Thompson HJ (2022). A systematic review of virtual reality therapeutics for acute pain management. Pain Manag Nurs.

[25] Farashi S, Bashirian S, Jenabi E, Razjouyan K (2024). Effectiveness of virtual reality and computerized training programs for enhancing emotion recognition in people with autism spectrum disorder: a systematic review and meta-analysis. Int J Dev Disabil.

[26] Corrigan N, Păsărelu CR, Voinescu A (2023). Immersive virtual reality for improving cognitive deficits in children with ADHD: a systematic review and meta-analysis. Virtual Real.

[27] MacHale R, Ffrench C, McGuire B (2023). The experiences and views of adults with intellectual disabilities accessing digital mental health interventions: a qualitative systematic review and thematic synthesis. J Appl Res Intellect Disabil.

[28] Valentine AZ, Brown BJ, Groom MJ, Young E, Hollis C, Hall CL (2020). A systematic review evaluating the implementation of technologies to assess, monitor and treat neurodevelopmental disorders: a map of the current evidence. Clin Psychol Rev.

[29] Oh SS, Moon JY, Chon D (2022). Effectiveness of digital interventions for preventing alcohol consumption in pregnancy: systematic review and meta-analysis. J Med Internet Res.

[30] Whittingham LM, Coons-Harding KD (2021). Connecting people with people: diagnosing persons with fetal alcohol spectrum disorder using telehealth. J Autism Dev Disord.

[31] Munn Z, Peters MDJ, Stern C, Tufanaru C, McArthur A, Aromataris E (2018). Systematic review or scoping review? Guidance for authors when choosing between a systematic or scoping review approach. BMC Med Res Methodol.

[32] Tricco AC, Lillie E, Zarin W (2018). PRISMA Extension for Scoping Reviews (PRISMA-ScR): checklist and explanation. Ann Intern Med.

[33] Hong QN, Pluye P, Fabregues S (2018). Mixed Methods Apprisal Tool (MMAT) version 2018. http://mixedmethodsappraisaltoolpublic.pbworks.com/w/file/fetch/127425851/MMAT_2018_criteria-manual_2018-04-04.pdf.

[34] Hong QN, Fàbregues S, Bartlett G (2018). The Mixed Methods Appraisal Tool (MMAT) version 2018 for information professionals and researchers. Education for Information.

[35] Aromataris E, Munn Z (2020). JBI Manual for Evidence Synthesis.

[36] Coles CD, Strickland DC, Padgett L, Bellmoff L (2007). Games that "work": using computer games to teach alcohol-affected children about fire and street safety. Res Dev Disabil.

[37] Coles CD, Kable JA, Taddeo E, Strickland DC (2015). A metacognitive strategy for reducing disruptive behavior in children with fetal alcohol spectrum disorders: GoFAR pilot. Alcohol Clin Exp Res.

[38] Hanlon-Dearman A, Edwards C, Schwab D, Millar MC, Longstaffe S (2014). “Giving voice”: evaluation of an integrated telehealth community care model by parents/guardians of children diagnosed with fetal alcohol spectrum disorder in Manitoba. Telemed J E Health.

[39] Hundert AS, Huguet A, Green CR (2016). Usability testing of guided internet-based parent training for challenging behavior in children with fetal alcohol spectrum disorder (Strongest Families FASD). J Popul Ther Clin Pharmacol.

[40] Kable JA, Coles CD, Strickland D, Taddeo E (2012). Comparing the effectiveness of on-line versus in-person caregiver education and training for behavioral regulation in families of children with FASD. Int J Ment Health Addict.

[41] Kable JA, Taddeo E, Strickland D, Coles CD (2016). Improving FASD children's self-regulation: piloting phase 1 of the GoFAR intervention. Child Fam Behav Ther.

[42] Louw JG, Olivier L, Skeen S, van Heerden A, Tomlinson M (2019). Evaluation of a custom-developed computer game to improve executive functioning in 4- to 6-year-old children exposed to alcohol in utero: protocol for a feasibility randomized controlled trial. JMIR Res Protoc.

[43] McCoy SW, Hsu LY, Jirikowic T, Price R, Ciol M, Kartin D (2015). Sensorimotor training to affect balance, engagement, and learning for children with fetal alcohol spectrum disorders. Physiotherapy.

[44] McCoy SW, Jirikowic T, Price R (2015). Virtual sensorimotor balance training for children with fetal alcohol spectrum disorders: feasibility study. Phys Ther.

[45] Padgett LS, Strickland D, Coles CD (2006). Case study: using a virtual reality computer game to teach fire safety skills to children diagnosed with fetal alcohol syndrome. J Pediatr Psychol.

[46] Petrenko CLM, Kautz-Turnbull CC, Roth AR (2021). Initial feasibility of the “Families Moving Forward Connect” mobile health intervention for caregivers of children with fetal alcohol spectrum disorders: mixed method evaluation within a systematic user-centered design approach. JMIR Form Res.

[47] Petrenko CL, Parr J, Kautz C, Tapparello C, Olson HC (2020). A mobile health intervention for fetal alcohol spectrum disorders (Families Moving Forward Connect): development and qualitative evaluation of design and functionalities. JMIR Mhealth Uhealth.

[48] Turner K, Reynolds JN, McGrath P (2015). Guided internet-based parent training for challenging behavior in children with fetal alcohol spectrum disorder (Strongest Families FASD): study protocol for a randomized controlled trial. JMIR Res Protoc.

[49] Gibbs A, Flanagan J, Gray L (2024). An Australian online training and support program for caregivers of children and youth with fetal alcohol spectrum disorder: Families Linking with Families. J Intellect Dev Disabil.

[50] Gibbs A, Harrington S, Robinson C, Brooks C, Dedman C (2020). “Getting on With It”: a course by caregivers, for caregivers—pilot overview and evaluation report. https://www.nofasd.org.au/parents-carers-and-families/support-for-caregivers-families/.

[51] Price AD, Mukherjee RAS, Webster A (2023). Development and pre-feasibility testing of specific: a psychoeducation programme for caregivers of children with fetal alcohol spectrum disorder (FASD). J Child Fam Stud.

[52] Jirikowic T, Westcott McCoy S, Price R, Ciol MA, Hsu LY, Kartin D (2016). Virtual sensorimotor training for balance: pilot study results for children with fetal alcohol spectrum disorders. Pediatr Phys Ther.

[53] Gibbs A (2019). An evidence-based training and support course for caregivers of children with foetal alcohol spectrum disorder (FASD) in New Zealand. Adv Dual Diagn.

[54] Chu JTW, McCormack JC, Marsh S, Bullen C (2023). Knowledge, attitudes, and practices towards fetal alcohol spectrum disorder in New Zealand educators: an online survey. J Intellect Disabil.

[55] McCormack JC, Chu JTW, Wilson H, Rahman J, Marsh S, Bullen C (2023). Knowledge, attitudes, and practices towards fetal alcohol spectrum disorder in the New Zealand social and community sector: an online survey. J Intellect Disabil.

[56] Ens CDL, Hanlon-Dearman A, Millar MC, Longstaffe S (2010). Using telehealth for assessment of fetal alcohol spectrum disorder: the experience of two Canadian rural and remote communities. Telemed J E Health.

[57] Benoit T, Bowes C, Bowman N (2002). Telemedicine diagnosis for fetal alcohol syndrome—the Manitoba experience. Paediatr Child Health.

[58] Sethu N, Vyas R (2020). Overview of Machine Learning Methods in ADHD Prediction.

[59] Fodor LA, Coteț CD, Cuijpers P, Szamoskozi Ș, David D, Cristea IA (2018). The effectiveness of virtual reality based interventions for symptoms of anxiety and depression: a meta-analysis. Sci Rep.

[60] Fioriello F, Maugeri A, D’Alvia L (2020). A wearable heart rate measurement device for children with autism spectrum disorder. Sci Rep.

[61] Koumpouros Y, Kafazis T (2019). Wearables and mobile technologies in autism spectrum disorder interventions: a systematic literature review. Res Autism Spectr Disord.

[62] Zakerabasali S, Ayyoubzadeh SM, Baniasadi T, Yazdani A, Abhari S (2021). Mobile health technology and healthcare providers: systemic barriers to adoption. Healthc Inform Res.

[63] Cho S, Ensari I, Weng C, Kahn MG, Natarajan K (2021). Factors affecting the quality of person-generated wearable device data and associated challenges: rapid systematic review. JMIR Mhealth Uhealth.

[64] Cole AC, Adapa K, Khasawneh A, Richardson DR, Mazur L (2022). Codesign approaches involving older adults in the development of electronic healthcare tools: a systematic review. BMJ Open.

[65] Fischer B, Peine A, Östlund B (2020). The importance of user involvement: a systematic review of involving older users in technology design. Gerontologist.

