# Supplementary material for: Technologies for Supporting Individuals and Caregivers Living With Fetal Alcohol Spectrum Disorder: Scoping Review
Source: JMIR Ment Health. 2024 Jul 11;11:e51074. doi: 10.2196/51074 (PMC11259581; doi:10.2196/51074)
Supplement: Multimedia Appendix 1 [file mental-v11-e51074-s001.docx]

| Search number | Search terms |
| --- | --- |
|  |  |
| 1 | "Fetal alcohol spectrum disorders(s)" OR "fetal alcohol syndrome" OR "Foetal alcohol spectrum disorders(s)" OR "foetal alcohol syndrome" OR "foetal alcohol effects” OR “fetal alcohol effects" OR "alcohol related neurodevelopmental" OR "alcohol-related birth defects" |
| 2 | eHealth OR ehealth* OR e-health* OR “e health*” OR "electronic adj health" OR mhealth* OR m-health* OR "mobile health*" OR "m health" OR ePsych* OR e-Psych* OR eTherap* OR telehealth |
| 3 | gaming OR gamification OR videogam* OR “computer gam*” OR “video gam*” OR “electronic gam*” OR “virtual reality” OR “augmented reality” OR “interactive multimedia” OR “interactive software” OR “digital media” IR “software program*” |
| 4 | smartwatch* OR “wearable device*” OR wearables OR “real-time monitoring device*” OR actigraphy OR accelerometer* OR “wearable technolog*” OR “sensor technolog*” |
| 5 | app OR apps OR application OR smartphone* OR smart-phone OR mobile* OR phone* OR sensor* OR software OR “mobile technolog*” |
| 6 | “digital interventions” OR “digital technolog*” OR “interactive technolog*” |

n.b. searchers were restricted to those published since 2005 and English language only.
