# Supplementary material for: Technologies for Supporting Individuals and Caregivers Living With Fetal Alcohol Spectrum Disorder: Scoping Review
Source: JMIR Ment Health. 2024 Jul 11;11:e51074. doi: 10.2196/51074 (PMC11259581; doi:10.2196/51074)
Supplement: Multimedia Appendix 2 [file mental-v11-e51074-s002.docx]

Quality assessment tool (MMAT) applied to each included study

| **Study** | **Type of study** | **Approach** | **Methods** | **Findings** | **Interpretation** | **Coherence/adherence** | **Criteria Met** |
| --- | --- | --- | --- | --- | --- | --- | --- |
| Coles et al. [36] | Quantitative: randomised controlled trial | Yes | Yes | Yes | Yes | Yes | 5 |
| Coles et al. [37] | Quantitative: randomised controlled trial | Unclear | Yes | Yes | Unclear | Yes | 3 |
| Gibbs et al. [49] | Quantitative non-randomized studies | Yes | Yes | Yes | Yes | Yes | 5 |
| Gibbs et al. [50] | Mixed methods | Yes | Yes | Yes | Unclear | Yes | 4 |
| Hanlon-Dearman et al. [38] | Qualitative | Yes | Yes | Yes | Yes | Yes | 5 |
| Hundert et al. [39] | Mixed methods | Yes | Yes | Yes | Yes | Yes | 5 |
| Jirikowic et al. [52] | Quantitative: randomized controlled trial | Unclear | Unclear | Yes | Unclear | Yes | 2 |
| Kable et al. [40] | Quantitative: randomized controlled trial | Yes | Yes | Yes | Yes | Yes | 5 |
| Kable et al. [41] | Quantitative: randomised controlled trial | Yes | Yes | Yes | Yes | Yes | 5 |
| Louw et al. [42] | Study protocol |  |  |  |  |  | N/A |
| McCoy et al. [43] | Quantitative: randomized controlled trial | Unclear | Unclear | Yes | Unclear | Yes | 2 |
| McCoy et al. [44] | Qualitative | Yes | Yes | Yes | Yes | Yes | 5 |
| Padgett et al. [45] | Qualitative | Yes | Yes | Yes | Yes | Yes | 5 |
| Petrenko et al. [46] | Qualitative | Yes | Yes | Yes | Yes | Yes | 5 |
| Petrenko et al. [47] | Mixed Methods | Yes | Yes | Yes | Yes | Yes | 5 |
| Price et al. [51] | Mixed methods | Yes | Yes | Yes | Unclear | Yes | 4 |
| Turner et al. [48] | Study protocol |  |  |  |  |  | NA |
