# Supplementary material for: Technologies for Supporting Individuals and Caregivers Living With Fetal Alcohol Spectrum Disorder: Scoping Review
Source: JMIR Ment Health. 2024 Jul 11;11:e51074. doi: 10.2196/51074 (PMC11259581; doi:10.2196/51074)
Supplement: Multimedia Appendix 3 [file mental-v11-e51074-s003.docx]

Characteristics of studies included in the scoping review.

| **Study and County** | **Technology** | **Outcome of technology** | **Study method** | **Study participants and details** | **Study outcome** |
| --- | --- | --- | --- | --- | --- |
| Coles et al. [36] – United States | Computer game to teach fire and street safety | Fire and street safety for people with FASD | Pre and post intervention measures of street and fire safety skills | 32 children (aged 4-10 years) with fetal alcohol spectrum (FAS: a diagnosis with FASD) | Games could be effective at teaching safety skills |
| Coles et al. [37] – Georgia, United States | GoFAR program. 3 key components   1. GoFAR: Computer game for child with FASD – to teach self regulation and control 2. Parent training: Therapy sessions with parents to educate on child’s self regulation 3. Behavioural Analog therapy (BAT) to teach how to use learnt strategies in real word settings | Disruptive behaviour | Randomized controlled trial with three conditions   1. GoFAR program 2. FACELAND (provided instructions on emotional recognition in facial expressions), with parent training and BAT 3. Control Group | 30 children (aged 5-10 years) with FASD and their caregivers. | Favourable improvements in disruptive behaviour for those with FASD who participated in the GoFAR program. |
| Gibbs et al. [49] - Australia | Families linking with families   - 7 week program each session with different topic delivered via zoom | Support caregivers of those with FASD specific aims were to improve caregiver knowledge, provided caregivers strategies to implement ad develop peer-support relationships | Post-program questionnaire evaluation | 88 caregivers of children with FASD | Caregivers were satisfied with the program, had increased knowledge, developed skills and developed peer relationships. |
| Gibbs et al. [50] - Australia | Getting on with it (GOWI)   - 6 weeks program each session with a different topic delivered via zoom | Support caregivers of those with FASD specific aims were to improve caregiver knowledge, provided caregivers strategies to implement ad develop peer-support relationships | Post-program evaluation consisting of:   1. Brief questionnaire 2. Follow up telephone interviews | 12 caregivers of children with FASD | All caregivers were satisfied with the program and most felt they had a better understanding of FASD. Participants felt the zoom format was appropriate, but may not be suitable for all caregivers. |
| Hanlon-Dearman et al. [38] - Manitoba Canada | Telehealth assisted assessment and diagnosis; and support for rural families and those with FASD.   1. Assessment and diagnosis: locally conducted psychometrics and therapy then a telehealth consultation with experts 2. Support: follow up education and support provided to families | Diagnosis of FASD and follow up support | Qualitative interviews to evaluate families experience | 16 families, three had been through assessment and diagnosis and 13 with follow up support. 19 caregivers (15 female, 4 male, 3 birth, 13 adoptive or foster) | Families were satisfied with their telehealth experience. Telehealth helped build support networks and provided access to care that was not available due to living rurally, but some families did experience technology difficulties |
| Hundert et al. [39] - Canada | Strongest Families First: online (website-based) education program   - Contains 11 sessions each designed to teach a different parenting skill (e.g. preparing for change) - Each session contains: written material, interactive website, instructional video and audio clips. | Teaching parenting skills to those affected by FASD | Mixed methods to evaluate the usability of the program, with three steps   1. Beginning with a Think Aloud while using the program 2. Semi-structured interviews to provide feedback 3. Given access to the program for 10 days and then completed a questionnaire on content and usability | 18 participants including professionals and caregivers of those with FASD. 8 caregivers, 7 female, 1 male, 1 birth parent, 7 adoptive and foster parents. All caregivers had access to computers at home and at least average computer skills* | The program was viewed positively and easy to use, but individual preferences on content varied between participants. |
| Jirikowic et al. [52] - Unitied States | STABEL (details in McCoy et al.[43]) | Motor ability and balance for people with FASD | Randomized controlled trial with three groups. Assessments completed baseline, 1 week after intervention and 1 month after intervention.   1. Laboratory: completed 1-2 sessions per week for one month 2. Home: completed 1-2 sessions per week for one month 3. Control | 29 children (aged 8-15 years) diagnosed with FASD. | Favourable improvements in motor skills for those who used STABLE at home and in the laboratory environment |
| Kable et al. [40] - Atlanta, United States | Web-based parent education program to teach knowledge and improved behavioural regulation of caregivers of those with fetal alcohol syndrome (FAS) | Knowledge of neurodevelopmental functioning, advocacy, and behavioural regulation.  Behavioural regulation | Randomized controlled trial with three groups   1. In person workshop 2. Web-based information 3. Informational packet | 59 caregivers of children diagnosed with FAS or partial FAS. Children on average 6.72 years | All groups showed improvement in knowledge. Only in person workshop and informational packet showed improvements in child behaviour. |
| Kable et al. [41] – United States | GoFAR program (details in Coles et al.[37]) | Disruptive behaviour | Randomized controlled trial with three groups   1. GoFAR: received access to GoFAR 2. Faceland: computer game that focused on facial expressions associated with emotions 3. Control | 30 children (aged 5 -10 years) with a clinical diagnosis of FAS or partial FAS their caregivers | Acceptable and improvements in behaviour |
| Louw et al. [42] – South Africa | Computer based cognitive training program. Built upon the FARR game, a game with tasks that become increasingly difficult requiring more attention and focus to achieve | Executive functioning (specifically attention, inhibition, and working memory) of person with FASD | Study protocol for a randomised controlled trial | N/A | N/A |
| McCoy et al. [43] – United States | STABEL a virtual reality, where people must move through hoops while their standing surface changes. | Motor ability and balance for people with FASD | Randomised controlled trial with two groups   1. STABEL group: participated in the STABEL intervention 2. Control group: no intervention | 20 children (aged 7-14 years) with FASD | Favourable improvements in motor skills for those who used STABLE |
| McCoy et al. [44] – United States | STABEL | Motor ability and balance for people with FASD | Pre and post intervention measures of sensory attention and postural control | 11 children with FASD and 11 typically developing children (aged 8-16 years) | Favourable program by those who used and showed improvements in motor skills |
| Padgett et al. [45] – Georgia, United States | VR game based on the home fire safety program, designed to teach fire safety skills that are applicable to real world situations | Fire safety skills for people with FASD | Case studies | 5 children (aged: 4 – 7 years) with FAS | Could be effective to support the learning of fire safety skills |
| Petrenko et al. [46] – United States | Families moving forward connect app.  An app designed to support caregivers of people with FASD, includes learning modules, support forums and resources. | Support caregivers of those with FASD | Focus groups to evaluate experiences with the families moving forward app | 25 caregivers of children with FASD | Positively viewed by participants |
| Petrenko et al. [47] – United States | Families moving forward connect app | Support caregivers of those with FASD | Testing of app and interviews | 20 caregivers (of children 3 – 17 years) and 17 support providers | Caregivers valued the app particularly the support forums and resources |
| Price et al. [51] - United Kingdom | Salford parents and carers education course for improvements in FASD outcomes in children (SPECIFiC). Education program delivered online.   - Seven session education course delivered via audio conferencing | Support caregivers of those with FASD | Mixed methods to evaluate the acceptability and feasibility of the program.   1. Evaluation of each session 2. Questionnaires completed pre and post program 3. Semi-structured interviews after the course | 10 parents and caregivers of children recently diagnosed with FASD (children aged 5-10 years) | The program was viewed favourably by participants and provided recommendations to improve the program. |
| Turner et al. [48] – Canada | Strongest Families FASD. A web-based interactive program. | Upskill and reduce distress experienced by caregivers of those with FASD | Study protocol for randomised controlled trial with two groups   1. Strongest families first group: access to online strongest families first program and telephone coach 2. Control group: provided a reading list of FASD web pages | N/A | N/A |

Note: * only the views of caregivers were included in this review
